# Factors Influencing the Development of Mild Cognitive Impairment in Community-Dwelling People Aged 75 Years and Older

**DOI:** 10.3390/geriatrics6040104

**Published:** 2021-10-28

**Authors:** Akio Goda, Shin Murata, Kayoko Shiraiwa, Teppei Abiko, Hideki Nakano, Koji Nonaka, Hiroaki Iwase, Kunihiko Anami, Yuki Kikuchi, Jun Horie

**Affiliations:** 1 Department of Physical Therapy, Faculty of Health Sciences, Kyoto Tachibana University, Kyoto 607-8175, Japan; murata-s@tachibana-u.ac.jp (S.M.); shiraiwa@tachibana-u.ac.jp (K.S.); abiko@tachibana-u.ac.jp (T.A.); nakano-h@tachibana-u.ac.jp (H.N.); horie-j@tachibana-u.ac.jp (J.H.); 2 Department of Rehabilitation, Faculty of Health Sciences, Naragakuen University, Nara 631-8524, Japan; nonaka@naragakuen-u.jp (K.N.); anami@naragakuen-u.jp (K.A.); 3 Department of Physical Therapy, Faculty of Rehabilitation, Kobe International University, Kobe 658-0032, Japan; iwase@kobe-kiu.ac.jp; 4 Department of Rehabilitation, Mitsubishi Kyoto Hospital, Kyoto 615-8087, Japan; kickpt4018@outlook.com

**Keywords:** balance ability, community-dwelling, dementia, educational history, hypertension, mild cognitive decline, people aged 75 years and older

## Abstract

In Asia, including Japan, dementia incidence peaks in older adults over ≥75 years; it is therefore important to detect mild cognitive impairment (MCI), and prevent its onset in this age group. Our study hypothesized that physical and psychological status would be associated with MCI incidence among community-dwelling people aged 75 years and older. The study population comprised 291 such individuals. Participants with a Mini-Mental State Examination score of 28 or more were considered non-MCI, and those with a score of less than 28 and greater than 24 were considered to have MCI. Several other measures were also evaluated, including information about their current medical visits due to diseases, depressive symptom severity, various physical functions (trunk function, 30 s chair-stand test, one-legged stance test, timed up and go test time, 5 m walking time, grip strength, knee-extension strength, and toe-grip strength), and body composition (body fat and skeletal muscle mass). Participants suspected of having MCI had significantly shorter educational histories, higher rates of medical visits due to hypertension, and poorer balance abilities. The results suggest that these indices can be considered screening indicators for detecting MCI in people aged 75 years and older, which may be useful for planning intervention programs for this population.

## 1. Introduction

The number of dementia patients is increasing worldwide, because of which dementia is considered a global public health priority [1]. Japan, in particular, has the highest prevalence of dementia among the Organisation for Economic Cooperation and Development countries at 2.3% [2], and the disability risk of developing dementia among the elderly is approximately 50% or more [3]. The social costs of dementia have been increasing in recent years [4]; therefore, it is desirable to establish interventions to prevent the onset of dementia to reduce its impact. The World Health Organization has reported that the peak age of dementia onset in Asia, including Japan, is 75–84 years [5]. Therefore, it is important to take measures to prevent the onset of dementia in people aged 75 years and older.

Traditionally, dementia has been analyzed using a framework that divides it into normal health and dementia stages, which are bridged by an intermediate stage called mild cognitive impairment (MCI) [6]. MCI is a symptomatic pre-dementia stage on the continuum of cognitive decline that is characterized by objective impairments in cognitive function which are not severe enough to require help with normal daily activities [7]. Patients with MCI have a high rate of progression towards dementia within a relatively short duration [8]. Therefore, MCI is considered an important indicator for dementia prevention interventions [9].

Factors associated with the development of MCI in community-dwelling older adults include physical factors such as a history of physical inactivity, poor one-legged balance [10], and decreased ability to perform activities of daily living [11]; neuropsychological factors such as the presence of subjective memory impairment, abnormal verbal semantic fluency [10], and decreased mood and motivation [11]; and physiological factors such as decreased serum BDNF [12]. Furthermore, it has been reported that certain life activities (e.g., driving a car, using a map to go to unfamiliar places, reading books and newspapers, attending cultural courses, attending community meetings, participating in hobbies and sports activities, and working in the field or garden) may play an important role in the regression from MCI to a normal state [13]. However, no study has examined the effects of physical and psychological conditions on the development of MCI, focusing only on people aged 75 years and older who require more specific preventive measures. In this study, we hypothesized that physical and psychological conditions are related to the incidence of MCI among community-dwelling people aged 75 years and older. By testing this hypothesis, we aimed to clarify the relationship between MCI incidence, physical and psychological conditions, and modifiable factors associated with MCI incidence among community-dwelling people aged 75 years and older.

## 2. Materials and Methods

This study was a cross-sectional survey of community-dwelling older adults, administered between September 2014 and September 2019. Participants were recruited in Yasu City, Shiga Prefecture, Japan, between 2014 and 2019 through flyers distributed from June to August each year. These flyers explicitly stated that participation would be uncompensated. Data on participants’ gender, age, height, weight, body mass index (BMI), and educational history were collected, after which participants completed the Mini-Mental State Examination (MMSE) as a test for general cognitive function. In addition, information about their current medical visits due to diseases, such as hypertension, hyperlipidemia, diabetes, cerebrovascular disease, cardiovascular disease, and cancer was obtained from the participants. The eligibility criteria for our study were as follows:

(1) Older adults aged ≥ 75 years;
(2) Individuals not suspected to have cognitive decline, measured via an MMSE score ≥ 24 [14].

The exclusion criteria were as follows:

(1) Participation in this study in previous years;
(2) Past medical history of mental illness;
(3) Inability to complete the measurement of all items.

**Figure 1:**
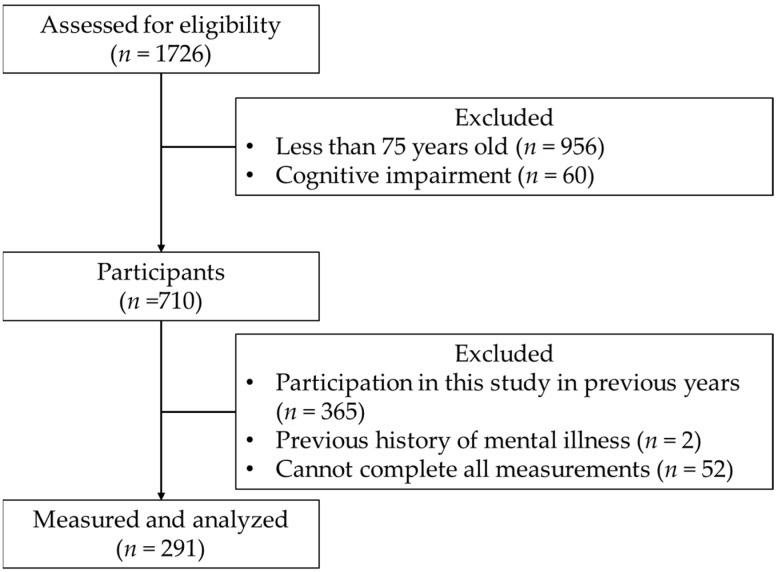
Flowchart of the selection process of the study participants.

Data from 291 participants were ultimately subjected to statistical analysis (Figure 1).

Written informed consent was obtained from each participant prior to participation. This study was conducted in accordance with the Helsinki Declaration and was approved by the Ethics Committee of Kyoto Tachibana University (accession nos. 14-5).

Several other parameters were assessed in addition to the participants’ general cognitive function. Depressive symptom severity was evaluated using a five-item version of the Geriatric Depression Scale (GDS-5). Furthermore, various physical functions (trunk function, 30 s chair-stand test (CS-30), one-legged stance test (OLST), timed up and go test (TUG) time, 5 m walking time, grip strength, knee-extension strength, and toe-grip strength) were measured. Finally, body composition (body fat and skeletal muscle mass) was measured.

Global cognitive function was assessed using the MMSE [15]. The MMSE is a short test that has extensively been used to assess cognitive functioning internationally. By covering 11 domains, from writing text to copying a drawing, this screening tool is both effective [16] and highly reliable [14]. The participants’ performance was evaluated based on their total MMSE score, which covered all of its measured domains. As in a previous study in which participants were classified as normal, MCI, or cognitively impaired based on MMSE scores, participants with a total score of 28 or more were considered as not having MCI [17], and those with a score of less than 28 and greater than 24 were considered to have MCI [18].

Depressive symptoms were evaluated using the GDS-5, a concise version of the GDS [19], which is a self-reported screening questionnaire created with a focus on the characteristic manifestations of depressive symptoms among older adults. This tool has been considered valid and reliable [20]. The GDS-5 consists of five questions in a yes/no format. Each positive response (i.e., indicating depressive symptoms) was awarded one point, with the sum of all items being used to assess the overall symptom severity.

Trunk function was assessed through performance in a sit-up test, which was conducted following the method described by Abe et al. [21]. Before commencing the test, participants lay in the supine position with both arms folded over their chest, knees bent at 90°, and feet together on the ground; their ankles were held by a technician. One sit-up was defined as lifting the upper torso until the elbows touched the thighs, and then returning to the initial position. The number of repetitions completed in 30 s was counted. Each participant was asked to perform a practice sit-up. If they were unable to do so, they did not proceed to the actual test; for these individuals, their performance was simply recorded as zero repetitions.

CS-30 was performed in accordance with the protocol described by Nakatani et al. [22]. Participants began the test seated upright in a chair that did not have armrests (height: 42 cm), with their arms folded across their chest. Before beginning the test, the researcher gave three verbal instructions: “Please keep your arms crossed over your chest,” “Fully straighten your knees when you stand up,” and “Please continuously repeat the exercise as quickly as you can.” The test consisted of repeatedly standing up and returning to the initial sitting position within 30 s, and the number of sit-to-stand cycles completed within 30 s was recorded.

Balance was evaluated using the OLST time. This test was performed according to the method described by MacRae et al. [23], but modified slightly for static balance. Participants were instructed to stand with their arms at their sides and to keep both eyes open, lift one foot off the ground, and keep it raised for as long as possible (maximum: 120 s). The measurement commenced when their foot was lifted off the ground and ended when the supporting leg moved or the suspended foot touched the ground, or once the maximum time was reached. The OLST was performed twice in total, once per leg; the longest time (of the two trials) was selected for analysis.

The TUG test was performed using the method described by Podsiadlo et al. [24]. Participants began the test seated upright in a chair without armrests (height: 42 cm). Once given the start signal, they were instructed to stand up from the chair, walk to a point 3 m away and back as quickly as possible, and then sit back down on the chair. The time taken to complete this task was recorded for the analysis.

The 5-m walking speed was assessed using the method described by Amano et al. [25]. The participants were instructed to walk 11 m in a straight line on level ground as quickly as possible. To eliminate potential confounders induced by acceleration near the start of the test and deceleration near the end, walking time was only measured for the middle 5 m segment (i.e., ignoring the first and last 3 m segments).

Grip strength was determined as described by Abe et al. [21]. Participants gripped a handgrip dynamometer (T.K.K.58401, Takei Scientific Instruments Co., Ltd., Niigata, Japan) as hard as possible, and grip strength was recorded. Grip strength of both the right and left limbs was measured, and the highest value was used for the analysis.

Knee extension strength was measured using the method described by Bohannon [26]. The participants began in a seated position, with hips and knees flexed at 90°. They were instructed to perform an isometric contraction (of the quadriceps) of one leg at maximal effort, pressing the lower shin near the ankle, against the sensor pad of a handheld dynamometer (µTasF-1, Anima Corporation, Tokyo, Japan). Four measurements were obtained in total, two each for the left and right legs. The maximum observed measurement was selected for analysis.

Toe-grip strength was measured using the method described by Souma et al. [27]. Participants began in a seated position with knees and hips flexed at 90°. One foot was placed over a toe-grip dynamometer (TKK 3362, Takei Scientific Instruments, Niigata Prefecture, Japan), with the handle positioned under the first proximal phalanx. Consistent toe position was ensured by sliding the device’s “heel stopper” to match the length of the foot. The participants were instructed to curl their toes at maximal effort. Four measurements were obtained in total, two each for the left and right foot; the maximum observed toe-grip strength was selected for analysis.

Body fat and skeletal muscle mass were measured using the bioelectrical impedance method with an InBody470 (InBody Japan Inc., Tokyo, Japan), as previously described in a study [28]. The participant stood on two metal electrodes and held a metal grip electrode. The body fat and skeletal muscle mass values were determined.

In the statistical analysis, we first checked normality for all demographic and assessment variables using the Shapiro–Wilk test. Next, these variables were compared based on the cognitive functioning status of the participants, i.e., MCI (28 > MMSE ≥ 24) and non-MCI (MMSE ≥ 28), using the χ^2^ test (gender, presence of medical consultation due to each disease), independent samples t-test (weight and BMI), and Mann–Whitney U test (other variables). Finally, the significance of the association between MCI incidence and each variable was examined using a logistic regression model (forced imputation), with the presence or absence of MCI incidence as the dependent variable (1: presence, 0: absence), the items that were significantly different between groups as the independent variables, and basic information (age, gender, and BMI) as the adjusted variables. SPSS Statistics software (version 26, IBM, NY, USA) was used for all analyses, and the significance level was set at 5%.

## 3. Results

The MMSE score of the study participants was 27.52 ± 1.90 points for the overall group, 25.78 ± 1.06 points for the MCI group, and 29.09 ± 0.82 points for the non-MCI group. Of the total participants, 138 (47.4%), i.e., approximately half, were diagnosed with MCI.

Table 1 summarizes the descriptive statistics for each assessment and the demographic variables. Data were compared based on the MCI determination estimated according to each participant′s MMSE score (i.e., MCI and non-MCI). Significant differences between the two groups were found in educational history, whether the participants had visited a medical institution due to hypertension, OLST, and TUG. There were no significant differences in other items between the two groups.

Table 2 shows the results of the association between MCI status and each index using the logistic regression model. The results of the univariate regression analysis confirmed significant associations with MCI incidence in education history, presence of medical consultation due to hypertension, OLST, and TUG. Next, the results of multivariate logistic regression analysis (forced imputation method), in which all items were imputed as independent variables, showed that education history (odds ratio (OR) = 0.87, 95% confidence interval (CI) = 0.77–0.97), medical visits due to hypertension (OR = 0.50, 95% CI = 0.30–0.84), and OLST (OR = 0.98, 95% CI = 0.98–0.99) were significantly related to MCI incidence.

## 4. Discussion

In this study, we hypothesized that physical and psychological status would be associated with MCI incidence among community-dwelling people aged 75 years and older. Participants with MCI had significantly shorter educational histories, lower rates of medical visits due to hypertension, and poorer balance abilities than participants without MCI did. Furthermore, logistic regression analysis identified a short educational history, no medical visits for hypertension, and a short one-legged standing time as independent factors associated with MCI incidence among community-dwelling people aged 75 years and older.

The incidence of MCI (28 > MMSE ≥ 24) was found in approximately half (47.4%) of the participants in this study. The incidence of MCI in this study was higher than the incidence of MCI (39.4%) in a previous study of community-dwelling elderly individuals aged 65 years or older [29]. This discrepancy in the results may be due to differences in the age range of the participants. In general, the proportion of people with cognitive decline increases with advancing age, but in this study, only people aged 75 years or older were included in the analysis, which may have resulted in a higher MCI incidence rate than in previous studies that included a younger population.

Statistical comparisons based on MCI status showed that participants with MCI had a shorter educational history, a lower percentage of medical visits due to hypertension, a shorter one-legged one-foot stand time, and a longer TUG time than those without MCI. Furthermore, these items were also significantly associated with the presence of MCI in the single regression analysis. Therefore, we conducted a logistic regression analysis by adding age, gender, and BMI as adjustment variables to the items that showed significant associations. The results showed that the factors clearly associated with MCI incidence were educational history, medical consultation for hypertension, and time spent standing on one leg with eyes open.

Previous studies have reported that a short period of education is a risk factor for cognitive decline [30]. Low education is associated with low health literacy [31,32], which is in turn associated with risk factors for cognitive decline, such as physical inactivity [33], unhealthy eating habits [34], metabolic syndrome [35], and other risk factors for cognitive decline [30,36]. Therefore, a shorter educational background may have influenced the development of MCI in the participants of the present study.

The results of this study, which showed a lower incidence of MCI in those who sought medical care due to hypertension, seem to contradict previous research showing that hypertension increases the risk of developing cognitive function [30]. This result may be influenced by the use of antihypertensive medications for the treatment of hypertension. The effect of medical treatment with antihypertensive drugs on improving cognitive function in elderly patients with hypertension has been reported [37]. In this study, we were not able to investigate the medication status of the participants, but it is likely that those who were aware of the symptoms of hypertension and visited a medical institution regularly were prescribed high-pressure medication [38]. Therefore, in the participants of this study, the incidence of MCI may have been lower in those who received medical care due to hypertension.

The results also suggest that impaired balance function (shortened one-legged standing time) in the MCI group affected the incidence of MCI. Decreased balance ability has been reported as a factor significantly identifying individuals with MCI [39]. This is also consistent with reports stating that impaired balance function begins to occur at the stage of MCI [40] and subjective cognitive decline [41]. Sabia et al. [42] reported that participants in the preliminary stages of dementia had lower physical activity levels compared to healthy participants. The MCI participants in this study may have also experienced a decline in balance due to a decrease in physical activity levels in the preliminary stages of dementia.

According to the results of this study, MCI in community-dwelling people aged 75 years and older was affected by educational history, medical visits due to hypertension, and balance function. Of these, one-legged standing time, which reflects balance function, is an index that can be easily performed in daily practice, being useful as a screening index for detecting MCI in the late elderly. In addition, visits to medical institutions due to hypertension and balance function are factors that can be modified through interventions, which may help prevent the onset of MCI in the late elderly. Therefore, based on previous reports of intervention studies, the following two methods can be applied to the prevention and management of dementia in MCI in the late elderly Japanese population:

(1) Improving health literacy through health education to increase the rate of healthcare visits [43] and appropriate blood pressure control [37,44]
(2) Low-impact physical interventions using tai chi [45] and yoga [46] to improve balance function in the elderly

This study has several limitations. First, because we adopted a cross-sectional design, we could not confirm the causality of the relationships found. Longitudinal studies are needed to clarify the relationship between changes in each index and the development of MCI. Second, we were not able to collect detailed information on the severity of disease and medication status of the participants in this study. In future, it will be necessary to consider this information to determine whether similar results can be obtained. Third, we were not able to measure factors previously reported as related to the development of MCI because of limitations in the number of personnel and available time for measurement. These factors should be assessed in future studies. Fourth, MCI status in this study was based on MMSE scores. Future studies should instead use well-developed clinical diagnostic criteria, such as objective memory impairment for age, a Clinical Dementia Rating of 0.5, and/or the absence of significant impairment in other cognitive domains (e.g., language, attention, abstraction, and orientation). Fifth, we did not measure the resting blood pressure levels of participants. Future studies should examine the effect of participants’ blood pressure control status on the development of MCI. Finally, the participants recruited for this study were relatively healthy. It is unclear whether the findings of this study are applicable to less healthy populations; thus, similar studies are needed to determine the same.

Despite these limitations, this study is significant because it suggests that in addition to the fixed factor of educational history, modifiable factors of seeking medical care due to hypertension (perhaps receiving medical treatment with antihypertensive drugs) and impaired balance ability have an effect on the development of MCI in the late older adults. The results of this study provide healthcare professionals with valuable information for the early detection of MCI and design of intervention programs for the prevention of dementia in this population.

## 5. Conclusions

This study examined the relationship between MCI incidence and basic information, medical visits, and physical and depression status in community-dwelling people aged 75 years and older. The results showed that MCI prevalence was related to educational history, medical visits for hypertension, and balance function. Therefore, geriatric medical professionals need to implement measures that target modifiable factors, such as promoting medical care for hypertensive patients and improving the balance function of older adults through physical interventions. Despite some limitations, the findings of this study may help health care providers in the geriatrics field to design and implement more effective intervention programs to prevent dementia in older adults.

## Figures and Tables

**Table 1:** Comparison of the fundamental information and measurements between the MCI and non-MCI groups.

Variable		MCI	Non-MCI	*p*-Value
		(n = 138)	(n = 153)	
Attribute	Age (years)	79.49 ± 3.67	78.79 ± 2.88	0.25
	Gender: Male/Female (*n*) *	42/96	47/106	1.00
	Height (cm)	152.5 ± 8.6	153.63 ± 8.5	0.25
	Weight (kg) ^☨^	52.26 ± 10.18	53.77 ± 8.94	0.18
	BMI (kg/m^2^) ^☨^	22.32 ± 3.16	22.76 ± 3.21	0.24
	Educational history (years)	10.9 ± 2.83	11.86 ± 2.3	< 0.01
Medical visits due to disease	Hypertension: yes/no (*n*) *	48/90	78/75	0.01
	Hyperlipidemia: yes/no (*n*) *	14/124	22/131	0.29
	Diabetes: yes/no (*n*) *	35/103	32/121	0.40
	Cerebrovascular disease: yes/no (*n*) *	19/119	21/132	1.00
	Cardiovascular disease: yes/no (*n*) *	10/128	19/134	0.17
	Cancer: yes/no (*n*) *	4/134	1/152	0.19
Mental and physical indicator	GDS-5 (score)	0.71 ± 0.96	0.63 ± 0.92	0.41
	Sit-up (repetitions)	5.95 ± 5.65	6.95 ± 6.08	0.20
	CS-30 (cycles)	17.2 ± 5.51	18.24 ± 5.02	0.05
	OLST (s)	21.95 ± 26.65	32.69 ± 33.62	< 0.01
	TUG (s)	6.8 ± 1.81	6.35 ± 1.3	0.04
	5-m walking time (s)	2.98 ± 0.95	2.83 ± 0.56	0.40
	Grip strength (kg)	25.41 ± 7.43	26.24 ± 6.83	0.19
	Knee-extension strength (kg)	18.14 ± 6.49	19.32 ± 6.39	0.15
	Toe-grip strength (kg)	8.42 ± 3.98	8.19 ± 3.64	0.74
Body composition	Body fat (kg)	14.64 ± 5.25	15.52 ± 5.83	0.31
	Skeletal muscle mass (kg)	19.96 ± 4.3	20.25 ± 3.96	0.31

Data are presented as mean ± standard deviation; MCI group: MMSE score 28 > MMSE ≥ 24; non-MCI group: MMSE score ≥ 28; Mann–Whitney U test; *: χ^2^ test; ☨: *t*-test using independent samples; MCI: mild cognitive impairment; BMI: body mass index; GDS-5: five-item version of the Geriatric Depression Scale; CS-30: 30 s chair-stand test; OLST: one-legged stance test; TUG: timed up and go test.

**Table 2:** Logistic regression analysis with the presence or absence of MCI as the dependent variable.

Variable		Univariate Analysis				Multivariate Analysis		
		95% CI for OR				95% CI for OR		
	OR	Lower	Upper	*p*	OR	Lower	Upper	*p*
Age (y)	1.07	0.99	1.15	0.07	1.01	0.93	1.09	0.90
Gender: Male	0.99	0.60	1.63	0.96	1.44	0.81	2.57	0.21
Female	1.00				1.00			
BMI (kg/m^2^)	0.96	0.89	1.03	0.24	0.96	0.88	1.04	0.30
Educational history (y)	0.86	0.78	0.95	<0.01	0.87	0.77	0.97	0.01
Medical visits due to hypertension: Yes	0.51	0.32	0.82	0.01	0.50	0.30	0.84	0.01
No	1.00				1.00			
OLST (s)	0.99	0.98	0.99	<0.01	0.99	0.98	0.99	0.04
TUG (s)	1.21	1.03	1.41	0.02	1.10	0.90	1.35	0.34

MCI: mild cognitive impairment; OR: odds ratio; CI: confidence interval; BMI: body mass index; OLST: one-legged stance test; TUG: timed up and go test.

## Data Availability

The datasets analyzed during this study are available from the corresponding author upon reasonable request.
